# Endometriosis and cumulative live birth rate after fresh and frozen IVF cycles with single embryo transfer in young women: no impact beyond reduced ovarian sensitivity—a case control study

**DOI:** 10.1007/s10815-019-01519-5

**Published:** 2019-07-16

**Authors:** Michael Feichtinger, Emelie Nordenhök, Jan I. Olofsson, Nermin Hadziosmanovic, Kenny A. Rodriguez-Wallberg

**Affiliations:** 1 Department of Reproductive Medicine, Division of Gynecology and Reproduction, Karolinska University Hospital, Novumhuset Plan 4, 141 86 Stockholm, Sweden; 2 Wunschbaby Institut Feichtinger, Vienna, Austria; 3 Department of Obstetrics and Gynecology, Division of Gynecologic Endocrinology and Reproductive Medicine, Medical University of Vienna, Vienna, Austria; 4 Department of Oncology – Pathology, Karolinska Institutet, Stockholm, Sweden; 5 Department of Women’s and Children’s Health, Karolinska Institutet, Stockholm, Sweden; 6 Uppsala Clinical Research Center, Uppsala University, Uppsala, Sweden

**Keywords:** Endometriosis, Cumulative live-birth, Frozen-thawed, SET, Cumulative pregnancy rate

## Abstract

**Purpose:**

To investigate the impact of symptomatic and surgically confirmed endometriosis on ovarian sensitivity index (OSI) and cumulative live-birth rates (LBR) using predominantly single embryo transfer (SET).

**Methods:**

Cross-sectional case-control study in a University-based ART program. Women with symptomatic and surgically confirmed endometriosis (*N* = 172), who underwent IVF/ICSI at Karolinska University Hospital were compared to controls without clinically suspected endometriosis (*N* = 2585). Two thousand seven hundred fifty-seven patients underwent 8236 treatment cycles (4598 fresh and 3638 frozen cycles). Primary outcome measures included Ovarian Sensitivity Index (OSI) estimated as collected oocytes/FSH dose and cumulative LBR/oocyte pickup (OPU). Generalized estimated equation (GEE) model accounting for dependencies between consecutive treatments were applied. Secondary outcomes included number of oocytes, pregnancy rate per OPU and per ET, LBR per ET, and miscarriage rate.

**Results:**

Patients diagnosed with endometriosis had significantly fewer oocytes collected (8.47 vs. 9.54, *p* = 0.015) and lower OSI (*p* = 0.011) than controls. There were no differences in cycle cancelations (*p* = 0.59) or miscarriages (*p* = 0.95) between the two groups. Cumulative LBR/OPU did not differ between women with endometriosis and controls (35.6% vs. 34.7%, respectively, *p* = 0.83). In both groups, more than 60% of women had consecutive FETs after fresh ETs (*p* = 0.49) with SET in > 70% of cases. The results were similar whether ovarian endometrioma was present or not.

**Conclusions:**

Our data support that a diagnosis of endometriosis, with or without present endometrioma, does not negatively affect ART cumulative results. The impact of endometriosis was discernible on OSI but not on clinical relevant outcomes including pregnancy and LBR.

## Background

Endometriosis is a benign but chronic inflammatory disease affecting around 10% of women of reproductive age with a high prevalence of up to 40% in infertile women [1]. Due to endometrial islets located outside the uterus, typical symptoms beyond infertility include dysmenorrhea, dyspareunia, and chronic pelvic pain [1]. The exact physiopathological processes and how endometriosis might impact female fertility remains elusive. There is no accordance in the literature for evidence of a different outcome of IVF/ICSI treatments in women with endometriosis, when compared to women without endometriosis. Recent observational studies suggest that isolated endometriosis may be associated with lower oocyte yield, but pregnancy and live-birth rates per transfer have been similar to those of controls [2]. Furthermore, this data is suggesting a detrimental effect of endometriosis only in combination with other infertility diagnoses, such as concomitant male factor infertility, diminished ovarian reserve, or tubal factor infertility. A meta-analysis has indicated a decrease in oocyte yields and reduced clinical pregnancy rate in patients with endometriosis undergoing IVF, compared to controls; however, multiple embryos had been replaced in most of the studies and the live-birth rate was similar [3, 4]. Although the pathway leading to fertility impairment in women with endometriosis is not yet completely understood, several endometriosis-associated factors such as reduced ovarian reserve, suboptimal oocyte quality, and unhealthy endometrial environment with negative impact on implantation rates have been discussed [5, 6]. Notably, the effect of age has been also difficult to disentangle in previous studies, as even in young women, the bulk of data on IVF/ICSI outcomes in women with endometriosis is based on treatments using multiple embryos for transfer [2, 7–9].

If one considers endometrial factors negatively influencing implantation as the predominant cause for reduced pregnancy rates in endometriosis patients, then such factors would likely lead to a reduced pregnancy rate regardless if the embryo transfers are using fresh or frozen thawed embryos. On the other hand, if ovarian factors are the main determinant, i.e., a reduced ovarian reserve, pregnancy and live-birth rates should not differ in fresh embryo transfer but cumulative pregnancy rates may be impaired due to reduced oocyte yield and supernumerary embryos obtained [10]. Therefore, we wished to investigate both the ovarian sensitivity and the cumulative live-birth rate of infertile women with surgical biopsy-confirmed, symptomatic endometriosis. We hypothesized that endometriosis would mainly affect ovarian factors and to a lesser extent would impair implantation. We tested our research question in a controlled cohort study design using infertile women with no signs of endometriosis as a control group.

## Methods

**Fig. 1:**
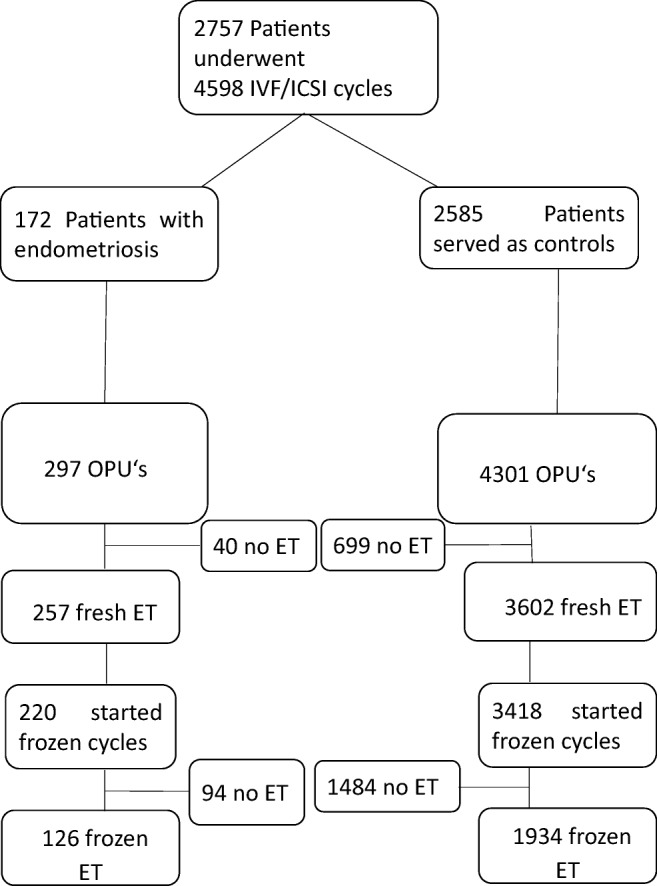
Flow-chart of the study cohort characteristics. OPU ovum pickup, ET embryo transfer

This study included 2757 infertile couples treated by IVF/ICSI at the Reproductive Medicine clinic of Karolinska University Hospital from January 2009 until December 2013 (Fig. 1). In Sweden, IVF/ICSI treatments are offered free of charge within the tax-funded healthcare system to couples with primary infertility and a woman’s age below 40 years. A policy of single embryo transfer (SET) is a current clinical routine, particularly in patients who are young and undergo their first IVF/ICSI attempt, after the evidence of a similar cumulative live-birth rate when compared to that after double embryo transfer (DET) and aiming at reducing maternal and perinatal complications associated to multiple conceptions [11]. At our center, SET is overall applied in 75% of treatments [12].

All patients included in this study were younger than 40 years of age and nulliparous at time of their first IVF/ICSI attempt. Exclusions were due to treatment using Preimplantation Genetic Testing (PGT), donor eggs, or if aimed at fertility preservation.

In the cohort, 172 women had histopathologically verified, symptomatic endometriosis after surgical biopsy [13] (cases) and 2585 women served as a reference population (controls without endometriosis). Patients were classified as minimal to mild endometriosis if the patient had undergone a diagnostic laparoscopy with findings of minimal to mild endometriosis and no history or present endometriomas. Patients were classified with moderate to advanced endometriosis if the patient had pelvic adhesions, deep infiltrating endometriosis or previous or present endometriomas at the start of her IVF/ICSI treatment.

The women underwent in total 8236 treatment cycles, 4598 fresh and 3638 frozen. Women with endometriosis underwent 297 fresh and 220 frozen-thawed cycles and the control women 4301 and 3405 cycles, respectively. The IVF/ICSI treatments were performed either in an GnRH agonist protocol or antagonist protocol, as described in detail elsewhere [12, 14]. During the study period, the clinical routine of our center included the transfer of cleavage embryos on day 2 in fresh cycles, and supernumerary good quality embryos were cryopreserved mostly at cleavage stage on day 2 or less frequently at blastocyst stage. Data on endometriosis were retrieved from the patient’s gynecology charts and relevant IVF/ICSI treatment data from the Reproductive Medicine’s electronic database (LinneFiler®, Fertsoft AB, Uppsala, Sweden), where data are prospectively collected and continuously updated after birth, with a ≥ 95% completeness.

## Statistical methods

Ovarian sensitivity was calculated as the ovarian sensitivity index (OSI), defined as the oocyte yield divided by the total dose of FSH administered × 1,000 [15, 16]. Owing to skewed distribution, the OSI was log-transformed and analyzed as a continuous variable. Live-birth rate by oocyte pickup including consecutive frozen-thawed cycles and OSI were set as primary outcome measures. Secondary outcome measures included pregnancy rate by ovum pickup, live-birth rate by embryo transfer, pregnancy rate by embryo transfer, miscarriage rate, and number of retrieved oocytes.

More than 60% of patients in our study had more than one embryo transfer (either fresh or frozen). Therefore, statistical analysis was done using a generalized estimated equation (GEE) model accounting for dependences between consecutive treatments [17]. With this method, the inherent weakness of a retrospective study—possible variable dependences—can be minimized and consecutive treatments analyzed. The model was applied for both categorical and continuous variables.

Categorical variables were displayed as counts and percentages, and continuous variables were displayed as means ± standard deviations (SD).

The analysis was carried out in SAS 9.4 (SAS-Institute, Cary, North Carolina, USA). The two-sided significance level was set to < 0.05.

The study was approved by the Regional Ethics Committee in Stockholm (Dnr 2011/1758–31/2).

## Results

Women with endometriosis did not differ in demographic variables including age (*p* = 0.91) or BMI (*p* = 0.81) (Table 1), when compared to controls. In women with endometriosis (*n* = 172), 38.4% had ovarian endometrioma (*n* = 66); and in 10.5% of cases (*n* = 18), endometriosis was associated with additional infertility factors. In the control group (*n* = 2585), 19.4% had male factor infertility (*n* = 502), 19.2% unexplained infertility (*n* = 497), 5.8% tubal factor (*n* = 150), 5.0% anovulation (*n* = 129), and 50.6% with combined factors or unspecified infertility (*n* = 1307). Following FSH stimulation, women with endometriosis showed a lower OSI (*p* <0.001) and smaller oocyte yields (*p* =0.004) (Table 2), corresponding to a proportion of 35.3% of low responders with endometriosis vs. 22.2% of controls. Only 6.5% of women were high responders in the endometriosis group vs. 15.9% of women in the control group. SET was performed in 72.0% of treatments in patients with endometriosis and 75.6% in controls, the small difference not being statistically significant (Table 2). Women with endometriosis received frozen-thawed embryo transfers in consecutive cycles in a similar proportion than controls (66.5%, vs 63.2%, respectively, *p* = 0.49), and the total number of ETs performed including fresh and frozen did not differ between the groups (*p* = 0.32). In fresh cycles, both the adjusted pregnancy rates per OPU and per ET did not differ between women with endometriosis vs. controls (32.4% vs. 31.1%, *p* = 0.73 and 35.8% vs. 34.6%, *p* = 0.81, respectively). The adjusted live-birth rates per OPU were also similar between the groups (26.4% vs. 25.5%, respectively, *p* = 0.78) and per ET (29.2% vs. 28.4%, respectively, *p* = 0.85).

**Table 1:** Comparison of patient characteristics and IVF/ICSI treatment cycle outcomes between women with endometriosis vs. controls. Data are mean or percent (95% CI)

		Women withendometriosis		Control women	*p* value
	Treatments (*n*)		Treatments (*n*)		
Age	297	33.59 (33.18–34.00)	4301	33.56 (33.44–33.68)	0.91
BMI	260	24.40 (23.90–24.91)	3666	24.31 (24.18–24.45)	0.81
Smoking	249	5.22%	3198	8.97%	0.10
Agonist protocol	297	81.14%	4226	56.70%	< 0.0001
ICSI	279	28.67%	3969	45.50%	< 0.0001
Treatments with embryo transfers	173	2.84 (2.53–3.16)	2585	2.68 (2.60–2.76)	0.32

*n*, number of treatments

**Table 2:** Comparison of IVF/ICSI treatment cycle outcomes between women with endometriosis versus controls. Data are mean or percent (95% CI)

	Women with endometriosis (treatments *n* = 297)	Control Women (treatments *n* = 4301)	*p* value	*p* value (adjusted for IVF/ICSI and agonist/antagonist)
Days of gonadotropin stimulation	10.85 (10.65–11.04)	10.47 (10.40–10.53)	0.0010	0.68
FSH total dose (IU)/OPU	2828 (2664–2992)	2370 (2328–2412)	< 0.0001	0.11
Oocytes (total number retrieved at OPU)	8.47 (7.86–9.08)	9.54 (9.37–9.71)	0.0040	0.0146
Ovarian sensitivity index	4.17 (3.75–4.60)	5.74 (5.59–5.90)	< 0.0001	0.0002
Embryos obtained	4.35 (3.90–4.81)	4.32 (4.19–4.46)	0.91	0.35
Embryoscore (1–10) day 2	9.50 (9.34–9.67)	9.41 (9.36–9.46)	0.29	0.33
Frozen embryos	2.24 (1.95–2.53)	2.51 (2.42–2.61)	0.13	0.11
Embryos to ET (fresh)	1.28 (1.22–1.34)	1.24 (1.23–1.26)	0.27	0.50
Embryos to ET (thaw)	1.10 (1.05–1.16)	1.08 (1.07–1.10)	0.49	0.50
SET (%)	71.98 (66.46–77.51)	75.62 (74.22–77.03)	0.25	0.48
Canceled cycles (no OPU or no ET) (%)	13.47 (9.56–17.37)	16.23 (15.13–17.33)	0.22	0.59
Clinical pregnancy/stimulation start (%)	30.98 (25.69–36.27)	28.95 (27.59–30.30)	0.47	0.73
Clinical pregnancy/OPU (%)	32.39 (26.92–37.87)	31.06 (29.62–32.49)	0.65	0.73
Clinical pregnancy/ET (%)	35.80 (29.90–41.70)	34.55 (33.00–36.11)	0.70	0.81
Clinical pregnancy/ET Thaws (%)	26.19 (18.41–33.97)	31.59 (29.52–33.67)	0.26	–
Cumulative clinical pregnancy/OPU (fresh and frozen ET) (%)	42.61 (36.82–48.39)	40.41 (38.89–41.93)	0.48	0.60
Cumulative clinical pregnancy/ET (fresh and frozen ET) (%)	47.08 (40.94–53.23)	44.24 (42.62–45.86)	0.40	0.54
LBRs/stimulation start (%)	25.25 (20.28–30.22)	23.79 (22.51–25.06)	0.57	0.78
LBRs/OPU (%)	26.41 (21.25–31.57)	25.52 (24.17–26.87)	0.75	0.78
LBRs/ET (%)	29.18 (23.59–34.78)	28.39 (26.92–29.87)	0.79	0.85
LBRs/ET thaws (%)	22.22 (14.86–29.58)	26.16 (24.20–28.12)	0.37	–
Cumulative LBRs/OPU (fresh and frozen ET) (%)	35.56 (29.96–41.16)	34.67 (33.20–36.15)	0.76	0.83
Cumulative LBRs/ET (fresh and frozen ET) (%)	39.30 (33.29–45.31)	37.89 (36.30–39.47)	0.66	0.76
Miscarriages (%)	18.48 (10.40–26.56)	17.83 (15.70–19.96)	0.88	0.95

*OPU* ovum pickup, *ET* embryo transfer, *SET* single embryo transfer, *LBR* live-birth rate

In frozen-thawed cycles, pregnancy rates were also similar between the endometriosis and control group (26.2% vs. 31.6%, respectively, *p* = 0.26), as well as live-birth rates (22.2% vs. 26.2%, respectively, *p* = 0.37). The frequency of twin pregnancies in women who achieved livebirths was low, 1.6% in women with endometriosis and 1.7% of controls.

The primary outcome measure, cumulative live-birth rate, including all consecutive fresh and frozen-thawed cycles, was similar and did not differ significantly between women with endometriosis vs. controls (35.6% vs. 34.7%, respectively, *p* = 0.83) (Table 2). The findings were also similar in a subgroup analysis restricted only to patients presenting with ovarian endometrioma (Table 3). When comparing women with minimal to mild endometriosis vs. moderate to advanced endometriosis, no differences in cumulative live-birth rates were found (41.9% vs. 35.4%, respectively, *p* = 0.43) and the OSI were also similar (5.3 vs. 4.8, respectively, *p* = 0.56).

**Table 3:** Comparison of IVF/ICSI treatment cycle outcomes between women with present ovarian endometrioma versus controls

	Women with ovarian endometrioma (treatments *n* = 115)	Control women (treatments *n* = 4301)	*p* value	*p* value (adjusted for IVF/ICSI and agonist/antagonist)
Days of gonadotropin stimulation	10.84 (10.55–11.13)	10.47 (10.40–10.53)	0.0164	0.93
FSH total dose (IU)/OPU	3081 (2815–3347)	2370 (2328–2412)	< 0.0001	0.0316
Oocytes (total number retrieved at OPU)	7.64 (6.67–8.61)	9.54 (9.37–9.71)	0.0009	0.0055
Ovarian sensitivity index	3.60 (2.94–4.26)	5.74 (5.59–5.90)	< 0.0001	0.0005
Embryos obtained	4.10 (3.43–4.78)	4.32 (4.19–4.46)	0.54	0.13
Embryoscore (1–10) day 2	9.51 (9.26–9.75)	9.41 (9.36–9.46)	0.42	0.48
Frozen embryos	1.86 (1.45–2.26)	2.51 (2.42–2.61)	0.0044	0.0042
Embryos to ET (fresh)	1.29 (1.20–1.38)	1.24 (1.23–1.26)	0.28	0.48
Embryos to ET (thaw)	1.11 (1.02–1.19)	1.08 (1.07–1.10)	0.60	0.48
SET (%)	70.59 (61.59–79.58)	75.62 (74.22–77.03)	0.26	0.44
Canceled cycles (No OPU or no ET) (%)	11.30 (5.43–17.18)	16.23 (15.13–17.33)	0.18	0.23
Clinical pregnancy/stimulation start (%)	30.43 (21.90–38.97)	28.95 (27.59–30.30)	0.73	0.97
Clinical pregnancy/OPU (%)	31.25 (22.53–39.97)	31.06 (29.62–32.49)	0.97	0.97
Clinical pregnancy/ET (%)	34.31 (24.94–43.68)	34.55 (33.00–36.11)	0.96	0.84
Clinical pregnancy/ET thaws (%)	32.14 (19.52–44.76)	31.59 (29.52–33.67)	0.94	–
Cumulative clinical pregnancy/OPU (fresh and frozen ET) (%)	46.43 (37.05–55.81)	40.41 (38.89–41.93)	0.22	0.21
Cumulative clinical pregnancy/ET (fresh and frozen ET) (%)	50.98 (41.11–60.85)	44.24 (42.62–45.86)	0.19	0.27
Live-birth rates (LBRs)/stimulation start (%)	24.35 (16.38–32.31)	23.79 (22.51–25.06)	0.89	0.94
LBRs/OPU (%)	25.00 (16.86–33.14)	25.52 (24.17–26.87)	0.90	0.94
LBRs/ET (%)	27.45 (18.64–36.26)	28.39 (26.92–29.87)	0.84	0.78
LBRs/ET thaws (%)	25.00 (13.30–36.70)	26.16 (24.20–28.12)	0.86	–
Cumulative LBRs/OPU (fresh and frozen ET) (%)	38.39 (29.25–47.54)	34.67 (33.20–36.15)	0.41	0.39
Cumulative live-birth/ET (fresh and frozen ET) (%)	42.16 (32.41–51.90)	37.89 (36.30–39.47)	0.38	0.46
Miscarriages (%)	20.00 (6.06–33.94)	17.83 (15.70–19.96)	0.75	0.83

Data are mean or percent (95% CI). OPU = ovum pickup, ET = embryo transfer, SET = single embryo transfer, LBR = Live-birth rate

As regards to comparison between endometriosis vs. other infertility diagnosis, analysis showed a significantly lower cumulative live-birth rate in patients with tubal factor infertility compared with endometriosis but no differences between endometriosis vs. male factor infertility, anovulation, and unspecified or unknown infertility cause (Table 4).

**Table 4:** GEE models comparing cumulative live-birth rates per OPU in women with endometriosis compared with other infertility diagnosis

	Odds ratio	95% CI	*p* value
Endometriosis	1.000	1.000–1.000	–
Male factor	0.943	0.707–1.258	0.6901
Anovulation	1.020	0.693–1.502	0.9196
Tubal factor	0.637	0.434–0.935	0.0213
Unspecified	1.051	0.786–1.405	0.7390
Unknown	0.973	0.735–1.287	0.8486

In multivariate analyses, live-birth rates per stimulation start, per OPU, and per ET, both crude and adjusted for age, IVF/ICSI, and type of simulation protocol used, were similar and did not differ significantly between women with endometriosis vs. controls (Table 5).

**Table 5:** Odds ratio (95% CI) for live-birth rate (LBR) in women with endometriosis vs. women without endometriosis

	Model I: crude	Model 2: age adjusted	Model 3: further adjusted for IVF/ICSI and stimulation protocol
LBR per stimulation start			
Fresh cycle	1.08 (0.82–1.43), *p* = 0.57	1.09 (0.83–1.43) *p* = 0.55	1.03 (0.78–1.36) *p* = 0.86
Fresh and thawed cycles	1.08 (0.84–1.39) *p* = 0.55	1.08 (0.85–1.39) *p* = 0.52	1.01 (0.78–1.31) *p* = 0.94
LBR per OPU			
Fresh cycle	1.05 (0.79–1.39) *p* = 0.75	1.05 (0.80–1.39) *p* = 0.72	1.03 (0.77–1.36) *p* = 0.86
Fresh and thawed cycles	1.04 (0.81–1.34) *p* = 0.76	1.05 (0.81–1.35) *p* = 0.73	1.01 (0.78–1.31) *p* = 0.94
LBR per embryo transfer			
Fresh cycle	1.04 (0.78–1.39) *p* = 0.79	1.04 (0.78–1.39) *p* = 0.78	1.01 (0.75–1.35) *p* = 0.96
Fresh and thawed cycles	1.06 (0.81–1.39) *p* = 0.66	1.07 (0.82–1.39) *p* = 0.64	1.02 (0.78–1.34) *p* = 0.89

## Discussion

The findings of our study show that although women with endometriosis present with a significantly decreased ovarian response, as indicated by the significantly reduced ovarian sensitivity index (OSI) in that group, the chances to pregnancy and live-birth through IVF/ICSI treatment are maintained and do not differ significantly to those observed in women without endometriosis. Moreover, fresh and cumulative live-birth rates were similar between the groups per simulation start, per OPU, and per ET, and both crude and adjusted for age, IVF/ICSI, and type of simulation protocol used.

Cumulative live-birth in SET with consecutive frozen-thawed ET has been suggested to be the most relevant outcome in assisted reproductive techniques [10]. Especially in women younger than 36 years of age, SET is broadly applied [18] and Sweden has the highest rates of SET procedures worldwide with more than 70% of all performed ART cycles [18]. Since endometriosis is often associated with infertility even in women of young age, and because of possible endometriosis-related obstetric complications such as preterm birth and obstetrical bleeding, these patients could particularly benefit from SET [19–23]. The practice of SET has been shown to reduce the number of pregnancy complications and neonatal morbidity and mortality with no impact on cumulative live-birth rates [11, 24–26]. Therefore, SET potentially may also reduce healthcare costs in comparison to double embryo transfer (DET), due to reduced neonatal morbidity [27].

The impact of endometriosis on IVF/ICSI outcome has been unclear in previous studies. A large study reported no differences in live-birth rates in subsequent IVF cycles in patients with endometriosis versus tubal factor. That study described lower pregnancy and live-birth rates in patients with endometrioma; however, multiple embryos could have been transferred, and the exact number of transferred embryos was not reported [7]. Our finding of significantly reduced cumulative live-birth rates in women with tubal factor compared to endometriosis-related infertility is in agreement with the previous analysis of the SART database study by Senapati et al., which indicated that women with endometriosis may present with higher live-birth rates compared to women with tubal factor infertility [2]. A recent meta-analysis did not find any impairment of pregnancy or live-birth rate when ovarian endometrioma was present [28], but also in most of the studies included in the meta-analysis multiple embryos were transferred.

Most women in our study had SET, and the proportion of patients receiving either DET or SET was similar in both the endometriosis and control groups, as well as the proportion of fresh or frozen-thawed ETs. Our study supports that even women with a present ovarian endometrioma do not have impaired cumulative pregnancy or live-birth rates per transfer.

Plausible reasons for a possible fertility impairment in endometriosis are numerous. Changes in follicular environment, ovarian reserve, endometrial receptivity, or even impact of endometriosis on oocyte genetics have been discussed [5, 6, 29–32].

In the present study, patients with endometriosis presented with reduced ovarian sensitivity index (OSI), thus suggesting a lower ovarian response [15]. However, the prevalence of reduced ovarian reserve in endometriosis patients remains unclear. Generally, women with endometriosis without a history of ovarian surgery do not have lower ovarian reserve parameters compared to control women, and women with unilateral endometrioma do not seem to have affected spontaneous ovulation rates [33, 34]. However, several studies confirmed lower oocyte yield in patients undergoing ART with present endometrioma [28]. While the impact of endometrioma on infertility treatment was recently questioned, ovarian surgery is on the other hand associated with deleterious impact on outcome of assisted reproduction [35, 36]. This correlation is most likely caused by the detrimental effect of surgery on ovarian reserve [9]. Due to these findings, a more restrictive surgical approach in management of ovarian endometriomas is suggested in women with infertility [35].

Notably, a reduced oocyte yield, on average one oocyte less, was found in patients with endometriosis in the present study. However, cumulative live-birth rates were maintained and importantly, numbers of cryopreserved embryos were similar to that of controls. These results suggest a predominant effect of endometriosis on oocyte yield rather than on endometrial environment. Previously, endometriosis has been associated with reduced oocyte quality and embryonic development [5, 37]. In the present study, however, no difference could be found in fertilization numbers and day 2 embryo scores in endometriosis vs. the control group. However, lower rates of frozen embryos in patients with ovarian endometrioma could point to an impaired embryonic development. Another study had reported lower fertilization rates in patients with endometriosis compared with patients with tubal factor but similar fertilization rates when compared to other infertility diagnosis [2]. Ovarian endometrioma on the other hand has been associated with impaired embryonic development [38]. In the present study, patients with endometrioma had a smaller number of frozen embryos, but no impact on cumulative live-birth. One previous study assessing the effect of endometriosis on cumulative live-birth rates after IVF/ICSI found poorer results in patients with severe endometriosis. However, only a small number of patients was investigated and multiple embryos had been transferred [8]. Different endometriosis phenotypes, distinguishing between peritoneal endometriosis, ovarian endometrioma, and deep infiltrating endometriosis on the other hand do not differ in their IVF outcome [36]. Both, endometriosis and control patients had high rates of frozen transfer cycle cancelations (Fig. 1). This is attributable due to the fact that frozen transfers have been predominantly performed in the natural cycle and therefore several transfers had to be canceled due to our weekday-only routine [39].

The present study consists of a sizable number of women undergoing sequential fresh and frozen ART cycles. The large number of cycles, prospectively entered data, and a high proportion of single embryo transfers represent the main strengths of our study. In Sweden, all public funded IVF/ICSI outcome data, including obstetric outcome gets reported compulsory—resulting in complete datasets with limited missing data.

To account for the interference between consecutive cycles and correlations between different outcomes, we applied GEE models. They minimize the negative impact of retrospective data analysis and allow for the analysis of consecutive treatment cycles. Thus, GEE models are most appropriate to evaluate cumulative live-birth rates [40]. Additionally, multivariable logistic regression models have been applied to adjust for differences between the study groups. In our study, we did not analyze different stages of endometriosis. The prevalence of endometriosis in our whole study cohort (6%) is low when compared to the described 25–40% prevalence of endometriosis in infertile populations [1]. This can be attributed to the fact that only women with symptoms and clinically evident endometriosis with histopathological confirmation by surgical biopsy were considered in our group of women with endometriosis, rather than asymptomatic sub-clinical or low grade endometriosis that might be underdiagnosed in the present study. Due to the absence of symptoms and thus no suspicion of endometriosis, most women in the control group did not undergo laparoscopy. Therefore, some of the women in the control group might have asymptomatic and undiagnosed endometriosis—a weakness of the present study that has to be acknowledged. However, analysis comparing cumulative pregnancy rates between women with diagnosed endometriosis in our study vs. women with several other infertility diagnosis did not reveal any differences between the groups, except for the analysis between endometriosis and women with tubal factor infertility, a finding that has been previously described by others [2]. To our knowledge, this is the largest study to date investigating cumulative live-birth rates, including frozen-thawed cycles in patients with endometriosis compared to a large cohort of infertile patients undergoing ART and receiving mostly single embryo transfer.

## Conclusion

Although a reduced ovarian sensitivity index was demonstrated in the group of patients with endometriosis, the practice of SET and consecutive frozen embryo transfer can be encouraged in this patients group, as the cumulative live-birth rate was not reduced in patients with endometriosis.
